# Single‐cell RNA sequencing reveals the effects of capsaicin in the treatment of sepsis‐induced liver injury

**DOI:** 10.1002/mco2.395

**Published:** 2023-10-05

**Authors:** Qian Zhang, Jing Liu, Jing Shen, Jinhuan Ou, Yin Kwan Wong, Lulin Xie, Jingnan Huang, Chunting Zhang, Chunjin Fu, Junhui Chen, Jiayun Chen, Xueling He, Fei Shi, Piao Luo, Ping Gong, Xueyan Liu, Jigang Wang

**Affiliations:** ^1^ Department of Critical Medicine, and Shenzhen Clinical Research Centre for Geriatrics Shenzhen People's Hospital First Affiliated Hospital of Southern University of Science and Technology Second Clinical Medicine College of Jinan University Shenzhen Guangdong China; ^2^ Institute of Basic Integrative Medicine ，School of Traditional Chinese Medicine, and School of Pharmaceutical Sciences Southern Medical University Guangzhou Guangdong China; ^3^ State Key Laboratory for Quality Ensurance and Sustainable Use of Dao‐di Herbs, Artemisinin Research Center, and Institute of Chinese Materia Medica China Academy of Chinese Medical Sciences Beijing China; ^4^ Department of Oncology Shenzhen People's Hospital The First Affiliated Hospital Southern University of Science and Technology Shenzhen Guangdong China; ^5^ Department of Physiology Yong Loo Lin School of Medicine National University of Singapore Singapore Singapore; ^6^ Department of Infectious Disease Shenzhen People's Hospital The First Affiliated Hospital Southern University of Science and Technology Shenzhen Guangdong China; ^7^ Department of Emergency Shenzhen People's Hospital The First Affiliated Hospital Southern University of Science and Technology Shenzhen City Guangdong Province China

**Keywords:** capsaicin, inflammation, scRNA‐seq, sepsis

## Abstract

Sepsis is a difficult‐to‐treat systemic condition in which liver dysfunction acts as both regulator and target. However, the dynamic response of diverse intrahepatic cells to sepsis remains poorly characterized. Capsaicin (CAP), a multifunctional chemical derived from *chilli peppers*, has recently been shown to potentially possess anti‐inflammatory effects, which is also one of the main approaches for drug discovery against sepsis. We performed single‐cell RNA transcriptome sequencing on 86,830 intrahepatic cells isolated from normal mice, cecal ligation and puncture‐induced sepsis model mice and CAP‐treated mice. The transcriptional atlas of these cells revealed dynamic changes in hepatocytes, macrophages, neutrophils, and endothelial cells in response to sepsis. Among the extensive crosstalk across these major subtypes, KC_Cxcl10 shared strong potential interaction with other cells when responding to sepsis. CAP mitigated the severity of inflammation by partly reversing these pathophysiologic processes. Specific cell subpopulations in the liver act collectively to escalate inflammation, ultimately causing liver dysfunction. CAP displays its health‐promoting function by ameliorating liver dysfunction induced by sepsis. Our study provides valuable insights into the pathophysiology of sepsis and suggestions for future therapeutic gain.

## Graphical abstract

Single‐cell transcriptome reveals the protective mechanism of capsaicin against sepsis induced liver injury in mice. The transcriptional atlas of cells revealed dynamic changes in hepatocytes (Hep), macrophages, neutrophils (Neu), and endothelial cells (Endo) in response to sepsis. Among the extensive crosstalk across these major subtypes, KC_Cxcl10 shared strong potential interaction with other cells when responding to sepsis.

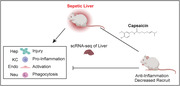

## 1 INTRODUCTION

Sepsis is a life‐threatening condition characterized by organ dysfunction caused by a dysregulated response to infection.^1^ The processes of inflammation imbalance and immune dysfunction are primarily involved in the pathogenesis of sepsis.^2^, ^3^, ^4^ Liver as a front‐line immune organ appears to be especially important, acting as not only a target of the host response but also a regulator of the inflammatory process.^5^, ^6^, ^7^ The symptoms of sepsis‐associated liver dysfunction (SALD) comprise hepatocellular injury, hypoxic hepatitis, and cholestasis.^6^, ^8^, ^9^ Even though extensive investigations on SALD pathophysiology have been performed, systematic, and in‐depth comprehension using traditional methods remains challenging, considering that intrahepatic cells are molecularly and functionally heterogenous due to gradients of nutrients, oxygen, hormones, and gut‐derived endotoxins in hepatic vessels.^10^, ^11^

Capsaicin (CAP), a major active compound of *Capsicum annuum L*., has been shown to harbor many pharmacological benefits including but not limited to antioxidant, anti‐inflammatory, and antilithogenic effects.^12^, ^13^ These beneficial effects, especially its anti‐inflammatory effect, make CAP a prospective candidate for sepsis therapy. Our previous studies have shown that CAP effectively inhibited sepsis‐related inflammation by decreasing the cytokine levels.^14^ Therefore, one of the main emerging strategies for drug discovery against sepsis is by antagonizing excessive released inflammatory factors and preventing cytokine storm formation in the early stages. The anti‐inflammatory activity of CAP has been illustrated with multiple mechanisms including the inhibition of proinflammatory factors IL‐6, TNF‐alpha, and nitric oxide production among others.^15^, ^16^

Single‐cell RNA transcriptome sequencing (scRNA‐seq) is revolutionizing the way we understand mechanisms underlying the development, homeostasis, and diseases of highly complex organs like the liver.^10^, ^17^, ^18^, ^19^ Through delineating the transcriptomic landscape and dynamic nature of cells with single‐cell resolution, scRNA‐seq technology can unravel the heterogeneity of hepatocytes (Hep) and nonparenchymal cells. In the mechanistic study of natural products, scRNA‐seq can comprehensively and accurately describe the differences in cell type and molecular status between physiologically normal tissues and pathological tissues before or after drug treatment, providing more information for the discovery of drug targets and pathways.^20^

In this study, we obtained scRNA‐seq data from normal, cecal ligation and puncture (CLP), and CAP‐treated mice, mapped the landscape of intrahepatic cells, and characterized the major cell populations and subtypes in response to sepsis. We further investigated the major cell–cell interactions between cell subsets. Our compendium of diverse intrahepatic cells responsive to sepsis would provide a valuable resource to understand the complexity of liver pathogenesis in sepsis, while unraveling the effects of CAP toward sepsis will pave the way for sepsis drug development.

## 2 RESULTS

### 2.1 Single‐cell RNA profiles the liver landscape of CAP‐treated sepsis model mice

**FIGURE 1:**
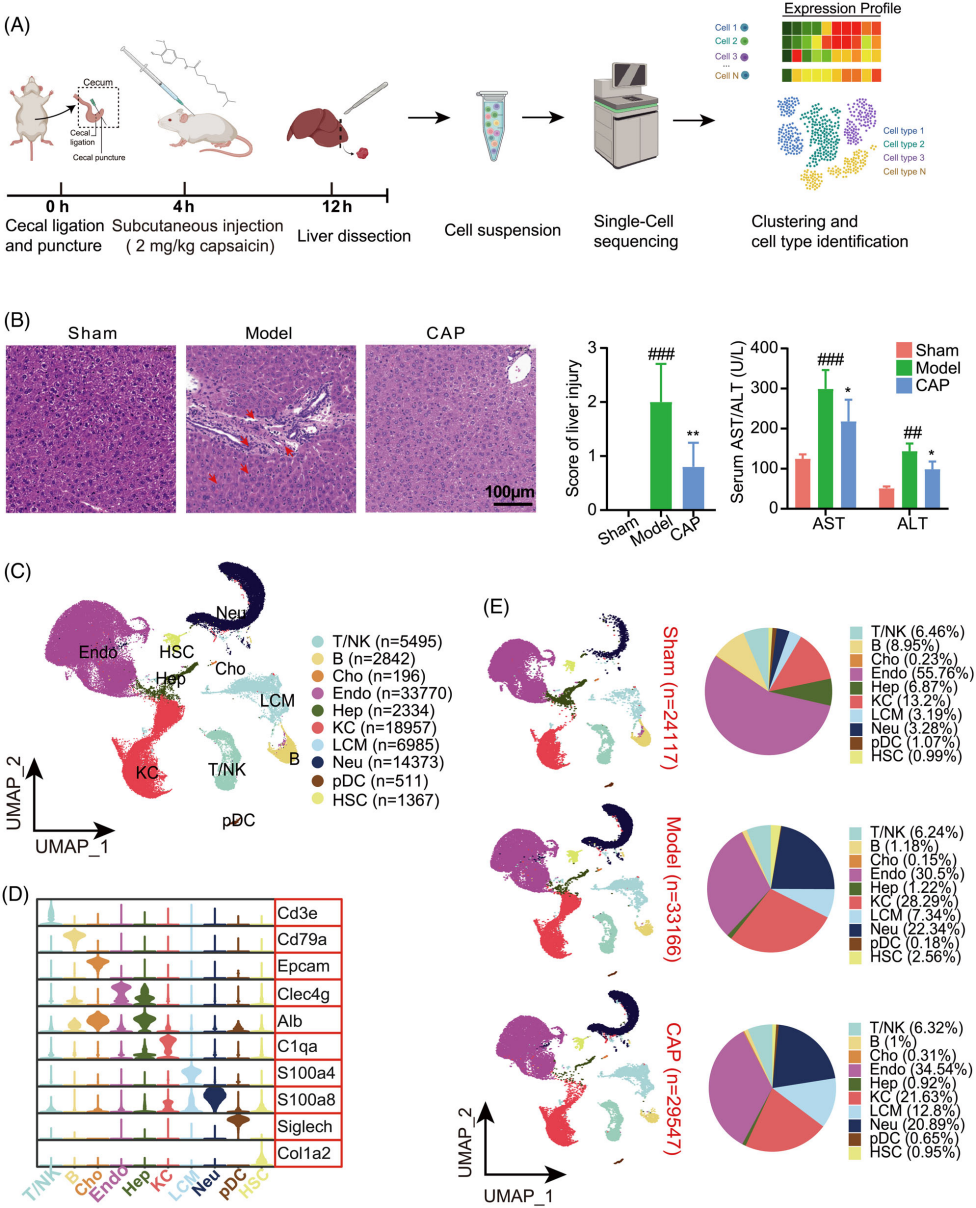
Single‐cell RNA profiles the liver landscape of capsaicin‐treated sepsis model mice. (A) Outline of the study design. (B) HE staining to validate dysfunctional livers and the serum AST or ALT, red arrows indicate inflammatory cell infiltration (mean ± SEM, *n* = 3; ##*p* < 0.01, ###*p* < 0.001 vs. sham, *p < 0.05, **p < 0.01 vs. model). (C) Uniform Manifold Approximation and Projection (UMAP) of 10×‐based single cells from liver, showing 10 clusters identified and colored by cell type. Each point corresponds to one single cell. (D) Violin plots showing the expression of signature genes across the 10 cell types. (E) Grouped UMAP plots and pie charts showing the proportions of ten clusters identified above across sham, model, CAP. CLP and CAP for cecal ligation and puncture and capsaicin treatment, respectively. (The same for all the following figures.) Figure1 was created with *BioRender.com*.

To characterize the cellular and molecular pathogenesis of sepsis and evaluate the effects of CAP, we established and employed CLP model mice. Where applicable, mice were treated with CAP at a dosage of 2 mg/kg (Figure 1A). Our previous research showed CAP had improved survival rate on CLP mice.^21^ According to HE staining and score of liver injury results, CLP mice showed significantly inflammatory cell infiltration and irregular cell proliferation, revealing the histological changes in the livers of septic mice. These histological changes were ameliorated by CAP treatment. Moreover, the levels of alanine transaminase (ALT) and aspartate transaminase (AST) in serum were notably reversed by treatment with CAP (Figure 1B).

Next, we obtained scRNA‐seq data to establish and study liver cell atlases from normal (sham), sepsis model (model), and CAP‐treated (CAP) mice, with three mice per group (Figure 1A). After quality control, we collected a total of 86,830 cells and identified 10 common major cell clusters, which correspond to T/NK (T/natural killer; *n* = 5495), B cells (*n* = 2842), Cho (cholangiocyte; *n* = 196), Endo (endothelial; *n* = 33770), Hep (*n* = 2334), KC (Kupffer; *n* = 18,957), LCM (liver capsular macrophages; *n* = 6985), Neu (neutrophils; *n* = 14,373), plasmacytoid dendritic cell (*n* = 511), and hepatic stellate cell (*n* = 1367), annotated with their signature gene expression (Figures 1C and D). To visualize the dynamic changes of clusters, we generated pie and column charts revealing comparable distribution patterns of these cell lineages across the three groups (Figures 1E and S1A–C). Compared with sham, significant fraction variations in the model group were observed in the Hep, Endo, macrophage, and Neu. Endo were reduced by approximately half while Hep cells dropped to around one‐sixth of baseline levels, indicating severe Endo damage and hepatocellular injury. Meanwhile, Neu and macrophages showed dramatic increases in proportion, which is suggestive of the proinflammatory condition elicited by sepsis. CAP could regulate the cellular profile of sepsis liver injury to a certain extent, mainly in that it could effectively reverse the changes of the intrinsic macrophage KC and Endo, also has a certain regulatory effect on Hep and Neu cells. Finally, as the majority of B cells were found to be immature, we did not incorporate B cells in the subsequent studies.

### 2.2 CAP inhibits inflammation in Hep of septic liver

**FIGURE 2:**
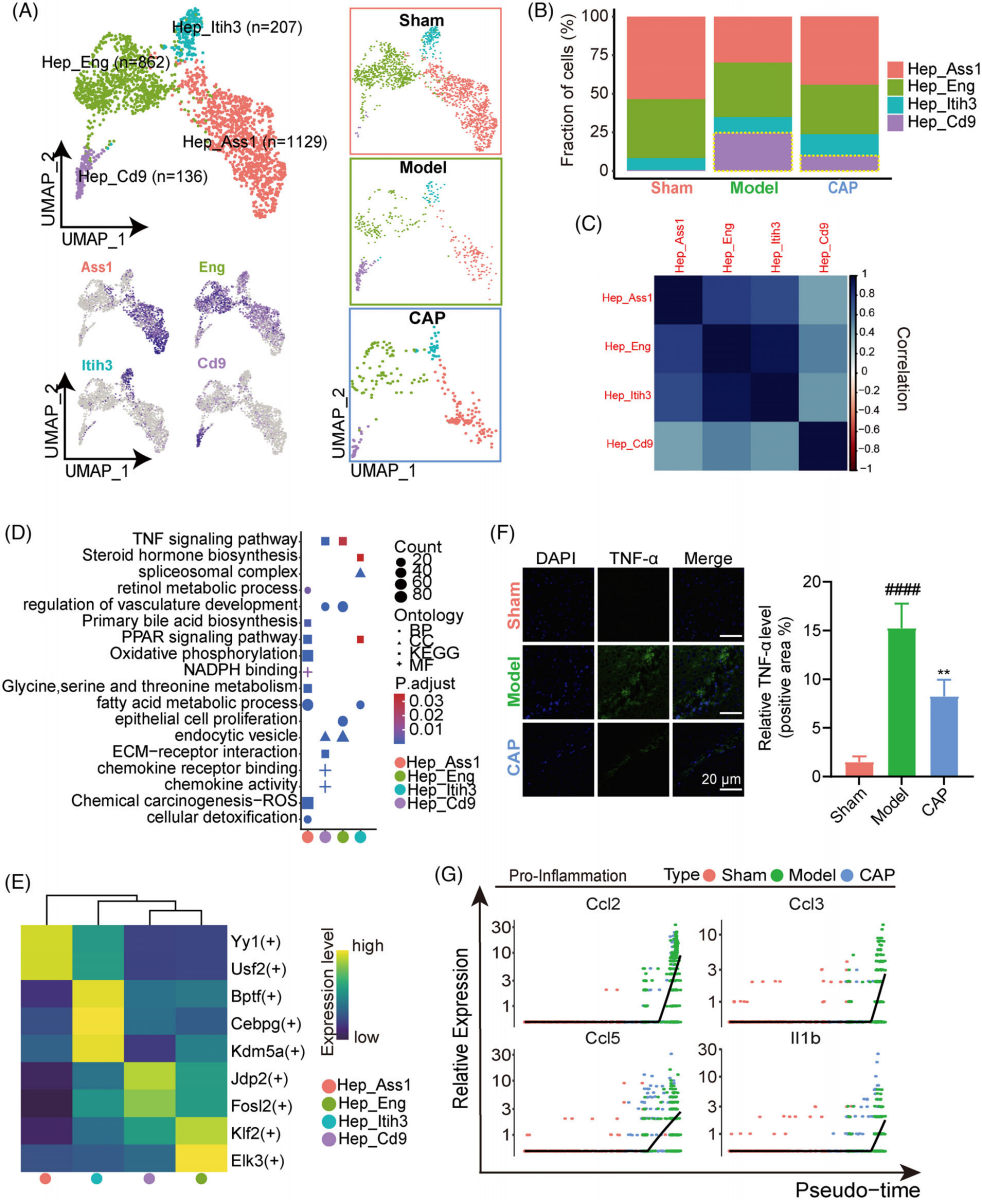
CAP inhibits inflammation in hepatocytes of septic liver. (A) UMAP plot for totally integrated (left top) or grouped (right) datasets, showing the major subpopulations of hepatocytes distinguished with different colors. The UMAP projection (left bottom) showing cell type annotation for hepatocyte subtypes by specific genes (Ass1, Eng, Itih3, Cd9). (B) Bar chart showing fractions of various cell subsets. (C) Heatmap showing Pearson correlation across the four cell types Hep_Ass1, Hep_Eng, Hep_Itih3, and Hep_Cd9. Colors shading from red to blue indicate growing values of Pearson correlation coefficient (PCC). (D) GO and KEGG enrichment of DEGs of each subpopulation compared with the other three subpopulations showing functional description. Dot size represents the significance and the shade of the color represents the adjusted *p* value. (E) Regulation heatmap based on AUC value shows signature transcription factors of each subset. (F) Immunofluorescence imaging of liver sections from mice of sham, model, and CAP using anti‐TNF‐α antibody, showing the expression of NF‐κB in hepatocytes (mean ± SEM, *n* = 3; ####*p* < 0.0001 vs. sham, **p < 0.01 vs. model). Scale bar, 20 mm. (G) The expression patterns of proinflammatory (left) and ROS‐related (right) genes in hepatocytes across sham, model, CAP along with the pseudotime.

We performed unsupervised clustering of hepatic parenchymal cells. A total of four clusters were identified, including Hep_Ass1, Hep_Eng, Hep_Itih3, and Hep_Cd9 (Figures 2A and S2A, B). Notably, the numbers of three subtypes (Hep_Ass1, Hep_Eng, and Hep_Itih3) were markedly reduced in the septic response whereas Hep_Cd9 exhibited a distinct tendency to accumulate in quantity. The rising tendency of Hep_Cd9 was also observed in its proportion, suggesting a special role in dysfunctional livers (Figure 2B). Strikingly, CAP reversed this increasing trend of Hep_Cd9 both numerically and proportionally (Figures 2A and B).

Meanwhile, Figure 2C showed *Pearson* correlations between Hep_Cd9 and any of the other subpopulations were weak, indicating the distinctive function of Hep_Cd9 in response to sepsis. The functional analysis for DEGs between each subtype suggested processes related to material metabolism such as fatty acid metabolic were enriched in cells of Hep_Ass1 and Hep_Itih3 whilst cells of Hep_Eng were enriched with epithelial cell proliferation and regulation of vasculature development. Hep_Cd9 however was significantly associated with proinflammatory changes with the enrichment of TNF signaling pathway, chemokine receptor binding and chemokine activity (Figure 2D). Analysis of transcription factor expression for the DEGs above revealed that Fosl2 and Jdp2, components of the AP‐1 transcription factor complex,^22^, ^23^, ^24^ were specifically and highly expressed in Hep_Cd9, further supporting its proinflammatory function in response to sepsis (Figure 2E). In contrast, Hep_Ass1 exhibited high expression of the transcription factor Yy1 which is deeply involved in metabolic disorders,^25^, ^26^ indicative of its role in metabolism.

To evaluate the anti‐inflammatory outcomes of CAP therapy, we detected inflammation‐associated factors including TNF‐α, mammalian target of rapamycin (mTORC1), and interferon‐α (IFN‐α) based on their corresponding module scores. Liver tissue sections were immunostained with TNF‐α, with results showing that inflammation was prevalent in all Hep from septic mice but markedly decreased in livers following CAP exposure, consistent with the changes of the TNF‐α score in hepatocyte subtypes from scRNA‐seq datasets (Figures 2F and S2C). CAP administration suppressed the score of inflammatory factors IFN‐α, one of the early activation genes in the septic response,^27^ while mTORC1 score (positive factors in the metabolic shift induced by hypoxia^28^) were significantly increased in the CAP group, suggesting a restorative effect on inflammatory factors in Hep (Figure S2C). Next, pseudotime analysis revealed that the transcriptional trajectory began with sham followed by CAP, and ended with model, indicating the effect of CAP on the hepatocyte state transition to the original normal status (Figure S2D). We next identified pseudotime‐dependent proinflammatory genes comprised of Ccl2/Ccl3/Ccl5/Il1b, all of which increased prominently at the termini of the trajectory where Hep of model were located (Figure 2G). CAP thus exerted its therapeutic effect by eliminating the general inflammation in Hep induced by sepsis.

### 2.3 CAP inhibits the proinflammatory response of KC_Cxcl10

**FIGURE 3:**
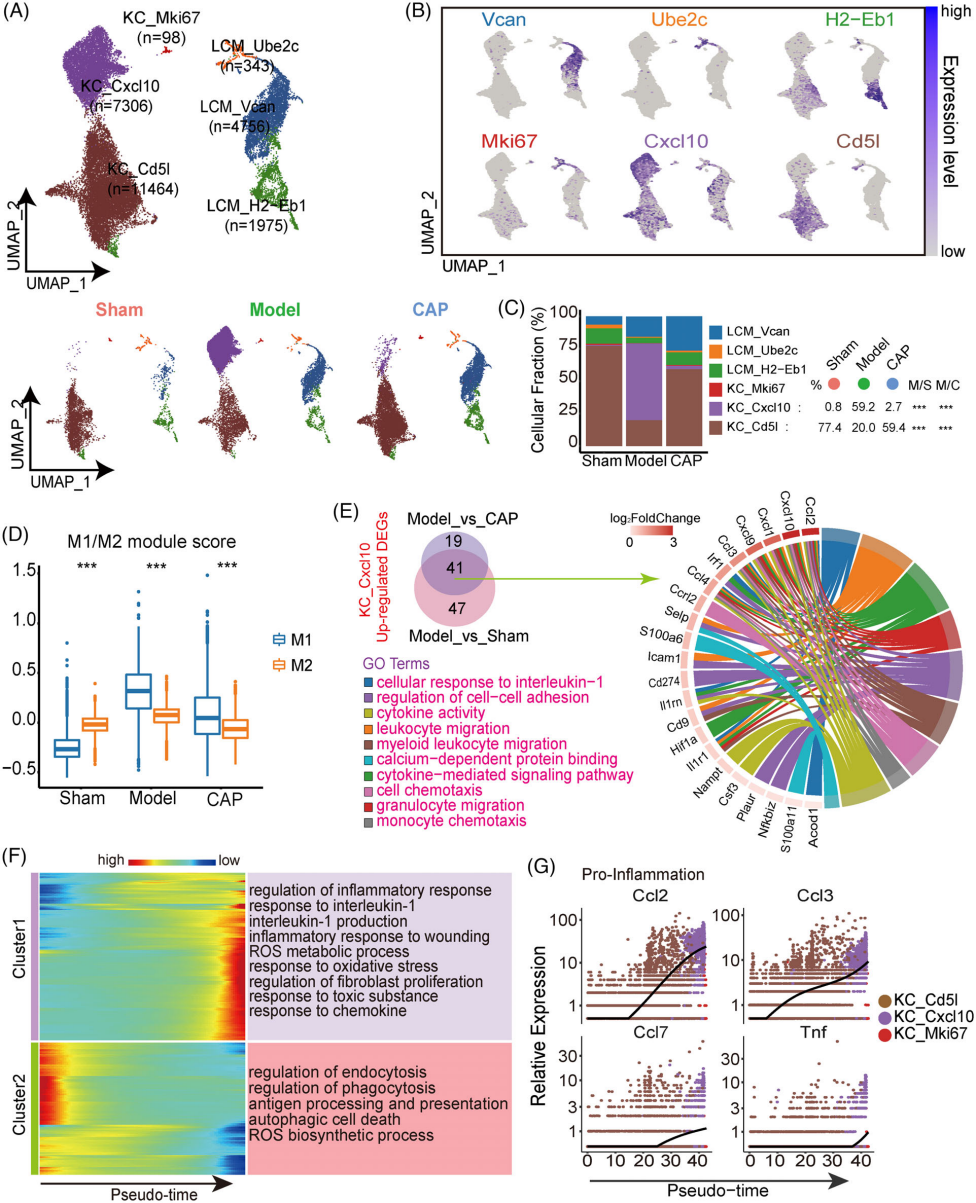
CAP inhibits the proinflammatory response of KC_Cxcl10. (A) UMAP plots for totally integrated (top) or grouped (bottom) datasets colored according to subtypes of macrophages. (B) UAMP plots showing characterization of macrophage subtypes with feature genes Vcan, Ube2c, H2‐Eb1, Mki67, Cxcl10, Cd5l. (C) Bar chart showing fractions of the six macrophage subsets across sham, model, CAP.M/S: model versus sham, M/C: model versus CAP. (D) Boxplot showing the M1/M2 (proinflammation /anti‐inflammation) expression score in sham, model, CAP to reveal the inflammatory status of overall macrophages. The whiskers indicate the data range. Groups were compared by wilcox‐test (***p < 0.001). (E) Venn Diagrams showing upregulated differential genes of KC_Cxcl10 from pairwise comparison (model vs. sham and model vs. CAP) and circle plot showing KEGG enrichment for common upregulated genes associated with chemotaxis. (F) Heatmap exhibiting different blocks of genes along the pseudotime trajectory and their GO/KEGG enrichment. (G) Dynamic expression patterns of proinflammatory genes along the pseudotime, including Ccl2, Ccl3, Ccl7, and Tnf.

To define major subpopulations of macrophages, we performed clustering and given rise to six clusters featured by their top ten most highly expressed genes (Figures 3A,B and S3A). Further proportion analysis revealed that the percentage of KC_Cxcl10 in model was dramatically increased compared with that in both sham and CAP, consistent with the variation in the concentration of CXCL10 protein expression evaluated by ELISA assay (Figures 3C and S3E). While CAP administration effectively reversed CLP‐induced theatrical expansion of the expression level of CXCL10 on tissue. This increase which was the largest among all compared subsets highlighted the potential capability of KC_Cxcl10 cells to respond to sepsis.

To investigate the relationship between KC_Cxcl10 and inflammation imbalance, we preferentially performed “classically activated” (M1) and “alternatively activated” (M2) scores on total macrophages^29^ based on gene sets. The increased M1/M2 value were observed in model, indicating an obvious state transition from anti‐inflammation to proinflammation compared with sham. CAP administration alleviated the severity of the inflammatory condition in overall macrophages with reduced M1/M2 value (Figure 3D). We executed DEGs analysis on KC_Cxcl10 between model and the other groups and enriched commonly 1062 downregulated DEGs, including receptor−mediated endocytosis (Figure S3B). Meanwhile, commonly 41 upregulated DEGs were enriched in the chemotaxis processes such as leukocyte migration and monocyte chemotaxis according to GO analysis (Figure 3E). Taken together, we propose that KC_Cxcl10 predominantly functioned as a regulator of chemotaxis of inflammatory cells, indicating a proinflammatory state consistent with the whole population. Correspondingly, CAP reversed the proinflammatory response of KC_Cxcl10 through instauration of these pathways and biological processes.

To further ascertain the role of KC_Cxcl10, we ordered overall KC (KC_Cxcl10, KC_Mki67, KC_Cd5l) into a pseudotime trajectory plot (Figure S3C). The development trajectory revealed that KC_Cxcl10 aggregated at the late period of the pseudotime with the prevalence of IL‐1 production, response to oxidative stress, response to chemokine, and with a decline of regulation of phagocytosis, regulation of endocytosis, antigen processing, and presentation (Figure 3F). These clues led us to assess the expressions of pro‐ and anti‐inflammatory cytokines. Elevated levels of the proinflammatory cytokine Tnf/Ccl2/Ccl3/Ccl7, as well as decreased anti‐inflammatory factors I10/Il1rn/Tgfβ1 and so on, were observed in KC_Cxcl10 (Figures 3G and S3D). Collectively, KC_Cxcl10 functioned as a proinflammation effector by regulating inflammatory cell chemotaxis.

### 2.4 CAP treatment suppresses the activation of neutrophil clusters

**FIGURE 4:**
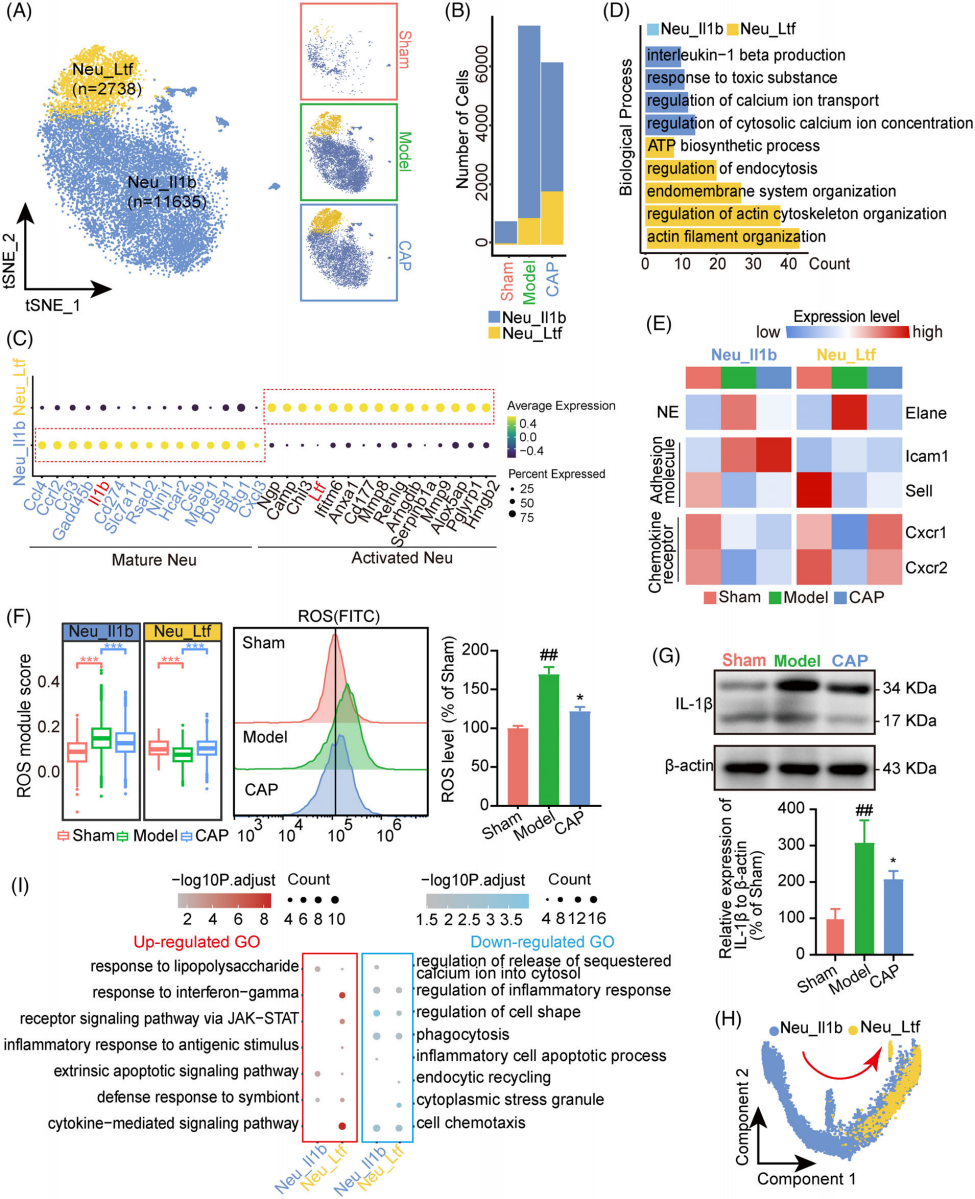
CAP treatment suppresses the activation of neutrophil clusters. (A) t‐SNE plots for totally integrated (left) or grouped (right) datasets colored according to subtypes of neutrophils. (B) Bar chart showing numbers of Neu_Il1b and Neu_Ltf across sham, model, CAP. (C) Plot showing expression of feature genes ranked top 15 in Neu_Il1b and Neu_Ltf. (D) Histogram showing GO analysis for DEGs of each neutrophil subtype. (E) Heatmap showing distinct expression patterns of selected genes between Neu_Il1b and Neu_Ltf across sham, model, and CAP. (F) Boxplot showing expression score of the proinflammatory factor ROS in Neu_Il1b and Neu_Ltf across sham, model, and CAP. The whiskers indicate the data range. Groups were compared by wilcox‐test (*p < 0.05, **p < 0.01, ***p < 0.001 vs. model, ##*p* < 0.01 vs. sham). (G) Western blot analysis showing IL‐1β protein expression in murine livers from sham, model, and CAP. (H) Pseudotime trajectory plot implemented by Monocle2 showing the developmental direction of neutrophil subtypes, labelled by the red arrowed line. (I) Bubble map showing GO enrichment for common DEGs of Neu_Il1b or Neu_Ltf obtained by pairwise comparison (model vs. sham, model vs. CAP).

We further divided liver Neu into two distinct clusters, Neu_Ltf and Neu_Il1b, were observed and characterized with corresponding signature genes (Figures 4A and S4A). Despite both subsets demonstrating similar accumulative trends in reacting to sepsis, the response of Neu_Il1b to CAP with decreased cell number was in contrast to the successive expansion of Neu_Ltf, indicating distinctions between Neu_Il1b and Neu_Ltf in response to CAP (Figure 4B). Differences between the two subsets were also indicated by their respective top 15 characterized genes (Figure 4C). In line with this expression pattern, DEG analysis revealed that certain genes pertaining to chemotaxis such as Ccl4, Ccl3, Ccrl2 were highly expressed in Neu_Il1b, suggesting that the Neu_Il1b subpopulation consisted of mature Neu (Figure S4B). In comparison, Neu_Ltf was characterized with high expression of Mmp8/Mmp9 (components of secretory granules^30^) and thus identified as activated Neu.

To further investigate the intrinsic distinctions between the two cell subtypes, we performed GO analysis for DEGs of the two subpopulations (Figures S4C and 4D). Neu_ Il1b was enriched with interleukin‐1 beta production while enrichment of regulation of endocytosis and regulation of actin cytoskeleton organization were observed in Neu_Ltf, suggesting their respective roles in cytokine generation and phagocytosis. Furthermore, the expression indicates that Neu_ Il1b exhibited a circulating phenotype of Neu with ICAM^low^Cxcr1^high^, but transformed to the reversely migrated Neu with ICAM^high^Cxcr1^low^ under septic circumstances,^31^, ^32^ suggesting the impairment of neutrophil migration in sepsis. This was further supported by the reduction of Sell favoring the rolling of Neu and Cxcr2 promoting neutrophil egress from bone marrow during sepsis^33^, ^34^ (Figure 4E). It has been reported that Neu in the process of reverse transendothelial migration showed a proinflammatory phenotype with increased superoxide expression.^35^ Thus we next examined the reactive oxygen species (ROS) score based on related gene sets in each subset and identified the level of ROS in three groups (Figure 4F). As predicted, Neu_ Il1b exhibited higher ROS scores in response to sepsis. Neu_Ltf with reduced ROS expression in sepsis may however be corelated with dysfunctional antimicrobial activity.^36^ Interestingly, abnormal ROS levels in both subtypes were clearly corrected by CAP exposure, indicating the beneficial effect of CAP which was further validated by decreased IL‐1b levels (Figure 4G).

To delineate the association between both subpopulations, developmental pseudotime analysis was applied showing a directional flow from Neu_Il1b to Neu_Ltf (Figure 4H). Notably, Neu_Ltf substantially accumulated at the end trajectory in sepsis which was then reversed to almost normal levels by CAP treatment (Figure S4D). The GO enrichment indicated pathways associated with response to INF‐gamma and cytokine‐mediated signaling pathway were enriched in commonly upregulated genes of Neu_Ltf, indicating that the excessive inflammation response was reversed by CAP to some extent. Likewise, reduced phagocytosis enriched in commonly downregulated genes of both subtypes was improved, suggesting its beneficial effect on impaired phagocytosis (Figure 4I).

### 2.5 CAP treatment alleviates the damage of Endo cells of septic livers

**FIGURE 5:**
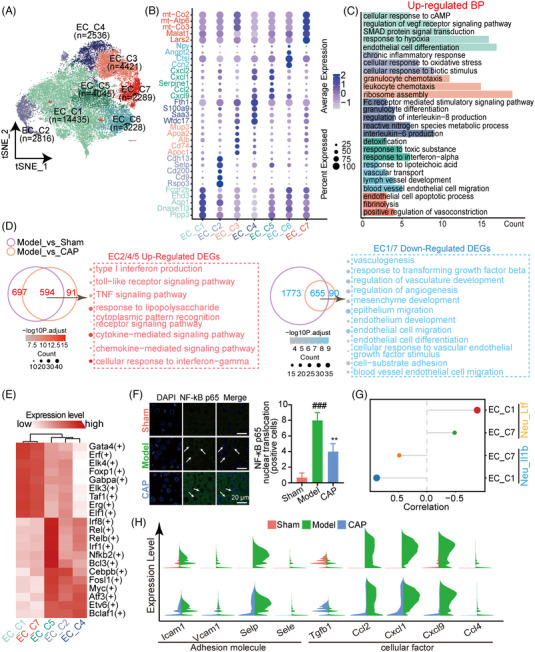
CAP treatment alleviates the damage of endothelial cells of septic livers. (A) t‐SNE plot for overall endothelial cells colored according to cell subtypes. (B) Bubble map showing expression of genes ranked top 5 from each endothelial cell subset. (C) Histogram showing GO analysis for DEGs of each endothelial cell subpopulation. (D) Venn Diagrams showing common upregulated DEGs of EC2/EC4/EC5 and commonly downregulated genes of EC1/E7 from comparison of model versus sham and model versus CAP. The arrowed line points to enrichment analysis for common DEGs. E2/4/5: EC_C2, EC_C4, and EC_C5; E1/7: EC_C1 and EC_C7. (E) Heatmap based on AUC value showing signature transcription factors of each subset EC_C2, EC_C4, EC_C5, EC_C1, and EC_C7. (F) Immunofluorescence images of liver sections from mice of sham, model and CAP, stained with anti‐NF‐κB antibody, showing the expression of NF‐κB in endothelial cells, white arrows indicate the location of NF‐κB (**p < 0.01 vs. model, ###*p* < 0.001 vs. sham). Scale bar, 20 mm. (G) Lollipop plot showing correlation between endothelial cell subsets (EC_C1, EC_C7) and neutrophil subsets (Neu_Il1b, Neu_Ltf). (H) Violin plots showing expression of selected genes primarily concerned with chemotaxis and adhesion in liver endothelial cells across sham, model, and CAP. ###*p* < 0.001 versus sham; **p < 0.01 versus model; *n* = 3.

We separated Endo cells into seven subpopulations, characterized by the top five genes in each subset (Figures 5A,B, and S5A). GO results shown that EC_C1 and EC_C7 were enriched with processes associated with Endo cell homeostasis such as Endo cell differentiation and positive regulation of vasoconstriction (Figure 5C). Meanwhile, EC_C2/EC_C3/EC_C4/EC_C5 were observed to have strong correlations with inflammation response. Next, by taking the logarithm of the model/sham and model/CAP ratio (Figure S5A), we observed quantities of EC_C2, EC_C4, and EC_C5 shared an increasing tendency in model compared with sham or CAP suggesting their similarity, so we classified them into one category (EC2/4/5) in the following analysis. Likewise, quantities of EC_C1 and EC_C7 were reduced in each comparison indicating endothelium damage, and we thus regarded EC1/7 as another classification responsive to inflammation and CAP.

GO analysis indicated that commonly upregulated proinflammation pathways such as TNF/cytokine‐mediated/chemokine‐mediated signaling pathways were broadly enriched in EC2/4/5 (Figure 5D). Taken together, a proinflammatory state was extensively represented across these subtypes. Conversely, CAP widely suppressed the inflammation response of these Endo cells. Notably, common downregulated DEGs of reduced subpopulations EC1/7 were dominantly enriched with Endo cell regeneration, indicating impaired vasculature in septic mouse livers and restoration of vasculogenesis with CAP treatment.

To further characterize the two categories of Endo cells, we performed transcription factor analysis, revealing a comparable expression pattern (Figure 5E). EC2/4/5 exhibited high expression of proinflammatory factors including Irf8, Rel, Relb, Irf1, Nfkb2, and Bcl3, as evidenced by immunostaining with nuclear factor Kappa‐B (NF‐κB) and indicating the role of EC2/4/5 in propagating the septic response (Figures 5E and F). Meanwhile, high expression of transcription factors (Gata4, Elk3, Taf1 and ERG) closely associated with Endo cell homeostasis was markedly observed in E1/E7,^37^, ^38^, ^39^, ^40^ leading us to investigate the association between E1/E7 damage with Neu. Strikingly, we identified a strong positive correlation between the proinflammatory subset Neu_Il1b and E1/E7, indicating that dysfunctional Neu_Il1b contributed to E1/E7 injury (Figure 5G). Comparing the expression of inflammatory genes between model and any other group revealed reduced expression of chemokines (Ccl2, Cxcl1, Cxcl9, Ccl4) and adhesion molecules (Icam1, Vcam1),^41^, ^42^ showing the anti‐inflammatory effect of CAP on Endo cells of septic livers (Figure 5H).

### 2.6 CAP treatment remodels intercellular crosstalk in the septic liver microenvironment

**FIGURE 6:**
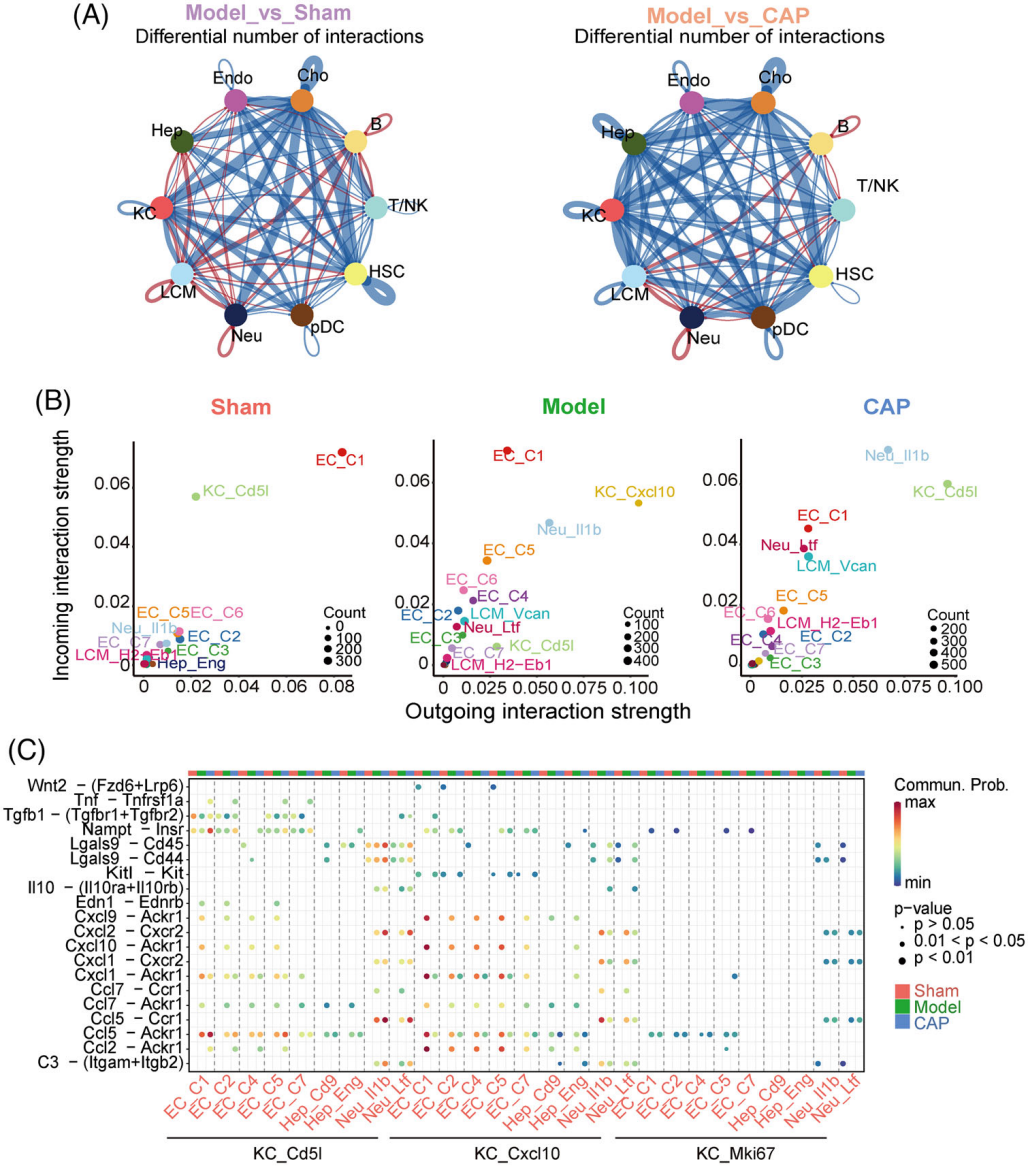
CAP treatment remodels intercellular crosstalk in the septic liver microenvironment. (A) Circle plots showing altered cell–cell communication network across major cell populations, inferred by LR pairing from DEGs of model versus sham or model versus CAP. Blue and brown lines indicate decreased and increased cellular interactions respectively. Each dot and color represent one cell type. The thickness of lines positively correlates with the LR number of cell–cell communication. The cells emitting the arrows express the ligands. (B) Bubble maps showing incoming and outgoing strength of cell subsets. (C) Each dot and color represent one cell type. Dot size is proportional to cell quantities in circle plots and LR numbers in the Bubble map. (D) Bubble map showing predicted ligand–receptor pairs between subsets of KCs and subsets of ECs, Hep, Neu across sham, model, CAP.

We investigate up‐ and downregulated interactions from differentially expressed LR pairs by comparing model with sham or CAP, showing that contact among the cell populations discussed above (including Endo, Hep, macrophages, and Neu) was notably enhanced in septic livers (Figure 6A). To investigate the central cell subpopulations shaping liver fate during sepsis, we mapped the interactions between the major cell subsets and calculated the incoming and outgoing interaction strengths (Figures 6B and S6B). In the model group, KC_Cxcl10 emerged as one core member of the network, harboring strong and extensive connections with Hep, Neu, and Endo, which was almost reversed to normal levels by CAP administration. Significantly, CAP drove the restoration of potent bidirectional interaction between KC_Cd5l and other cells which is attenuated in septic livers, indicating a beneficial effect on regulating cellular communication.

To elucidate the function of crosstalk from KCs to dominant subpopulations, we investigated significant LR pairs. Of the LR pairs between KC_Cxcl10/KC_Cd5l with Neu subpopulations, CC chemokines delivered by KC_Cxcl10/KC_Cd5l were commonly discovered, indicating their function in regulating neutrophil chemotaxis in the septic response (Figure 6C). In addition, we observed broad interactions between KC_Cxcl10/KC_Cd5l and Hep/Endo through chemokines (including Cxc and Cc subfamilies) and the receptor Ackr1 in response to sepsis. Given the function of atypical chemokine receptor Ackr1 in controlling chemokine levels and localization, we posit that KC_Cxcl10/KC_Cd5l could play a central role in the control of chemotaxis in the septic response. Additionally, CAP administration weakened the interaction from KC_Cxcl10 to Neu/Endo/Hep through chemokine‐receptors, suggesting its beneficial function on amplified inflammation.

## 3 DISCUSSION

Inflammation imbalance plays a predominant role in sepsis‐induced liver injury.^2^, ^43^, ^44^ Nevertheless, the pathogenesis of this excessive inflammation process remains incompletely illustrated on livers damaged by sepsis. Here, we applied scRNA‐seq to delineate a cell atlas of normal mice and CLP‐induced mice with or without CAP treatment and systematically unveiled pathological changes and the proportion of various cell subsets including Hep, macrophages, Neu and Endo cells, while the proportion of these cell subsets tended to be in the normal group after CAP treatment.

Hep coordinate an adaptive response of inflammation and metabolic shift by producing diverse acute‐phase proteins and cytokines during sepsis to achieve host defense and protection.^43^ In this study, we found a special subpopulation of hepatocyte subtype Hep_Cd9, which exhibits proinflammatory function involved in TNF signaling pathway and chemokine activity. Hep_Cd9 highly expressed transcription factors Fosl2 and Jdp2, suggesting it might activate regulation of chemokine production through the AP‐1 signaling pathway.^45^, ^46^ Additionally, Hep_Ass1 is a class of cell subtype with high expression of arginine metabolic enzyme Ass1 and transcription factor Yy1.^47^ Ass1 and Yy1 were responsible for the metabolic shift and adaption through PPAR signaling pathway.^48^ In particular, the inflammation response and the adaptive metabolic shift could be counterbalanced by CAP. CAP has been reported to exert a hepatoprotective property under septic conditions by mainly inhibiting oxidative and inflammatory processes.^49^

Macrophages show broad diversity and plasticity.^50^ KCs and LCMs are two key intrahepatic macrophages that secrete proinflammatory cytokines and perform phagocytosis after infection.^51^ In the present study, we identified three KC subpopulations and three LCM subpopulations, among which a KC subpopulation KC_Cxcl10 was characterized with dramatic expansion in septic livers but reduced by CAP. Further analysis revealed that chemokine and interleukin‐1 production was upregulated in KC_Cxcl10 subpopulation a proinflammatory regulator of chemotaxis. Previous research has been reported that KC induces intrahepatic Endo cell injury by producing Cxcl10, which leads to intrahepatic inflammatory response upon infection.^52^ Importantly, CAP reversed the proinflammation state and counteracted cytokine production from KC_Cxcl10, demonstrating the anti‐inflammation effect of CAP on macrophages. Furthermore, it has been observed that elevation of chemokines/cytokines (Ccl2, Ccl3, Ccl7, and Tnf) and decrease of anti‐inflammatory cytokines such as Il10 and Tgfβ were concomitant with the occurrence of KC_Cxcl10, the subset appeared to be proinflammatory cells primarily by releasing chemokines to regulating chemotaxis, thus expand inflammatory response.

Neu produce proinflammatory mediators, degranulation, and forming neutrophil extracellular traps.^53^ However, Neu are recruited during liver infection.^54^ Here we found that CAP induced partial repression of the number of overall Neu that substantially accumulated during sepsis. It is noteworthy that the subpopulation Neu_Ltf continued to expand after CAP exposure, reflecting the distinction between Neu_Ltf and Neu_Il1b. Based on the enrichment of upregulated DEGs of Neu_Il1b represented by interleukin‐1 beta production and Neu_Ltf by regulation of endocytosis. Thus, Neu_Il1b and Neu_Ltf may be professional in producing Il1b and mediating phagocytosis respectively. Migration and antimicrobial activity of Neu have previously been reported to be impaired during sepsis.^55^, ^56^ In our study, we found Neu_Il1b with Icam1^high^ Cxcr1^low^, =a proinflammatory phenotype based on the increased expression of ROS‐related gene, accounts for the damage in the endothelium and other organs.^56^, ^57^, ^58^ In contrast, reduced expression of ROS signature genes in Neu_Ltf suggested impaired antimicrobial function through ROS during sepsis, thus contributing to failure in preventing bacterial dissemination. Particularly, the efficacy of CAP on reversing the dysfunction of Neu and alleviating the degree of inflammation could also be inferred from the restoration of ROS levels in each subtype and reduced overall generation of Il‐1b.

Upon sensing pathogens, Endo cells reprogram toward a proapoptotic, proinflammatory, proadhesive, and procoagulant phenotype, but constant phenotypic changes can lead to Endo injury.^59^, ^60^ In this study, CLP‐induced sepsis mice showed Endo damage with significant reduction of total Endo cells, which was improved in the CAP group. Further analysis revealed that CAP ameliorated pathologically proinflammatory conditions of Endo cells through the NF‐kB signaling pathway and secretion of chemokines Ccl2/Ccl4/Cxc1Cxc9. Additionally, the proadhesive phenotype was also reprogrammed to the normal state based on the observation of low expression of adhesion molecules Icam1/Vcam1/Sele/Selp after CAP exposure.

Substantial inflammatory mediators are released during sepsis,^61^ determining intricate intercellular communications in the liver. As predicted, boosted interaction across the four populations discussed above was observed in model, among which KC_Cxcl10 in model showed the most remarkably enhanced crosstalk which was subsequently attenuated by CAP. Consistent with the regulation of cell chemotaxis found in Figure 3, KC_Cxcl10 communicated with subpopulations of Endo/Hep through the chemokine receptor Ackr1 (supported by computational paring), indicating the regulatory function of Endo/Hep on chemokine production and delivery by KC_Cxcl10. Simultaneously, enhanced interaction between KC_Cxcl10 and Neu subtypes through chemokine ligands and receptors such as Ccl5–Ccr1 indicated increased chemotaxis from KC_Cxcl10 to Neu during sepsis. Taken together, it appears that mutual regulation among KC_Cxcl10, Endo cells, Hep and Neu could escalate the inflammation response into a cytokine storm during sepsis. These results demonstrate that CAP rewires cell–cell communication crosstalk in sepsis and decreases immuno‐inflammatory responses.

There are some limitations in the present study. First, just as we use the original CAP to give mice subcutaneous injection, which can stimulate the skin mucosa of mice. In the future, we can reduce the irritation of CAP by using nanotechnology and biochemistry in order to achieve better therapeutic effect. Second, we observed a special cell subtype, KC_Cxcl10, whose chemotactic activity and activation play a key role in immune‐inflammation and phagocytosis, but the clonal relationship and signal network involved in regulation of KC_Cxcl10 were not explored. Therefore, other techniques such as gene editing techniques, chemotactic experiments, flow cytometer and spatial transcriptomics might be beneficial in revealing the intricate mechanisms of CAP's resistance to infection.

**FIGURE 7:**
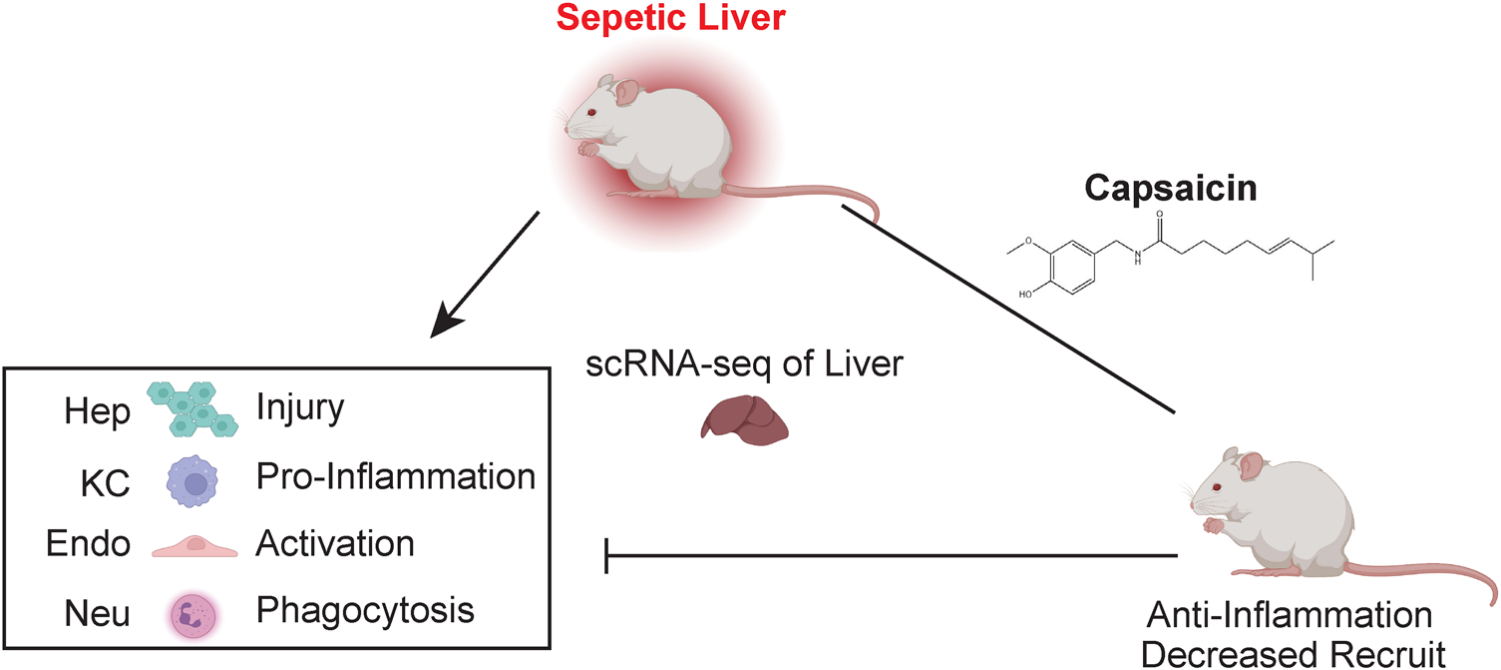
Model illustrating the mechanisms of the potent pharmacologic effects of CAP on livers injured by excessive inflammation in septic mice. Figure7 was created with *BioRender.com*.

In conclusion, our comprehensive characterization of liver dysfunction in the CLP‐induced sepsis model revealed the inflammatory role of Hep, macrophages, Neu, and Endo cell subpopulations, and simultaneously explored the anti‐inflammatory efficacy of CAP in sepsis (Figure 7). Our datasets provided a resource for further exploration of sepsis pathophysiology and drug discovery.

## 4 MATERIALS AND METHODS

### 4.1 Animal Experiments

BALB/c male mice with ages between 6 and 8 weeks were obtained from Vatalriver Laboratory (Guangdong, China). All animal experiments were approved by the Institutional Animal Care and Use Committee of Shenzhen People's Hospital.

### 4.2 CLP sepsis mouse model and treatment with capsaicin

CLP mice were established as previously described.^62^ In brief, mice were anesthetized via inhalation of 2.5% isoflurane and then 1% until the end of the CLP. The cecum was exposed, ligated, and then punctured twice with an 18‐gauge hypodermic needle. The cecum was then placed back to the peritoneal cavity, and the incision was closed, after which the mice were subcutaneously injected with 1 mL normal saline for resuscitation. A total of nine mice per group, three of which were used for scRNA‐seq experiments and six for molecular pharmacology experiments. Sham group underwent the same surgical procedures without ligation or puncture. Four hours later, CAP and other groups were subcutaneously injected with 2 mg/kg CAP solution and equivalent saline respectively. Liver samples of sacrificed mice were collected for scRNA‐seq analysis after 12 h.

### 4.3 Single cells processing from murine livers

Liver samples were cut into small pieces and enzymatically digested with the Mouse Liver Tissue Dissociation Kit (130‐105‐807) for about 30 min. The dissociated cells were filtered by a 70 mm strainer in the PBS to obtain uniform cell suspensions, followed by centrifugation at 300 g, 4°C for 10 min. After the supernatant was removed, the pelleted cells were suspended in Red Blood Cell Lysis Solution (130‐094‐183). After washing twice with PBS, the cell pellets were re‐suspended in sorting buffer (PBS supplemented with 0.04% bovine serum albumin) and processed into single cells.

### 4.4 scRNA‐seq libraries preparation and sequencing

scRNA‐seq libraries were established with the Chromium Next GEM Single Cell 3′ Kit v3.1 (10× Genomics), following the user's instructions. Briefly, the concentration of single cell suspensions was adjusted to about 1000 cells/μL. About 10,000 cells were captured in droplets to generate nanoliter‐scale Gel beads in EMulsion (GEMs) where cell lysis and barcoded mRNA occurred. After the mRNA was reverse transcribed in Appliedbiosystems (Thermo Fisher scientific), cDNA was isolated and purified from broken emulsions with Cleanup Mix containing DynaBeads and SPRIselect reagent (Thermo Fisher Scientific), followed by PCR amplification. For RNA‐seq library construction, amplified cDNA was successively fragmented and end‐repaired, double‐sided size‐selected, and PCR‐amplified with sample index primers. Constructed libraries were then purified and profiled for quality assessment. Single‐cell RNA was sequenced by an Illumina Hiseq 6000 sequencer with 150 bp paired‐end reads.

### 4.5 scRNA‐seq dataset processing

For quality control, raw data from each sample were sequenced with fastp (version 0.20.0) to filter low‐quality reads out from sequencing adapters and default Settings.^63^ Then, the raw gene expression matrix was generated using the Cell ranger (version 6.0.1) pipeline coupled with the mouse reference genome and analyzed by the Seurat R package (version 4.0.4) in R software (version 4.1.1).^64^ The matrix of the nine samples were integrated, and then normalized and scaled using Seurat's SCTransform function. Cells were further clustered based on an appropriate resolution. The algorithm of Uniform Manifold Approximation and Projection (UMAP) or t‐distributed stochastic neighbor embedding (t‐SNE) were utilized for two‐dimensional visualization.

### 4.6 Cell type annotation

The Seurat's FindAllMarkers function was conducted to distinguish expressed marker genes of each cluster. The cluster‐specific marker genes were identified to characterize the cell types. The procedures of principal components analysis, clustering and cell subtype annotation were performed for cell subtype identification as described above.

### 4.7 Differential expression and gene functional enrichment analysis

We identified differentially expressed genes between two groups of clusters using the Seurat's FindMarkers function. Genes meeting these criteria were considered as DEGs (min.pct = 0.25, *p*_val < 0.05, and avg_log2FC ≥ 0.25, min.diff.pct = 0.1). GO and KEGG analysis were performed using the clusterProfiler R package (version 3.18.1). *p* Values were calculated by the Hypergeometric test model and adjusted using BH. The biological process category was selected to profile the functional landscape, and visualized based on the number of enriched proteins and the adjusted *p* value (*Q* value) (*p* < 0.05).

### 4.8 Pseudotime analysis

The Monocle2 R package (version 2.20.0) was applied to infer the developmental trajectories of Hep and KCs. Seurat object was first converted to the CDS object, and then genes expressed with distinct alteration were determined by differential GeneTest function to evaluate the differential cell states. Next, plot_cell trajectory_function applying a dimensionality reduction to the data ordered the cells in pseudotime.

### 4.9 Cellular communication analysis

Cell–cell interaction network was deciphered by CellChat R package (version 1.1.3) based on evaluating the expression of ligand‐receptor pairs within cell populations.^65^ First, the normalized genes expression matrix and major cell types of three group mice acted as input for CellChat. CellChat then compared the differential number of interactions and interaction strength among mouse cell groups. The function circle plot was used to perform communication network visualization.

### 4.10 Western blot

Twenty micrograms of tissue‐proteins per sample were separated in SDS–PAGE gel and then transferred to previously activated PVDF membrane (Millipore). After blocking in 5% skim milk at room temperature, the membrane was incubated in order with primary antibody (IL‐1β, 26048‐1‐AP; Proteintech) at 4°C overnight and then with HRP‐IgG secondary antibody (HRP‐labeled Goat Anti‐Mouse IgG(H+L); Beyotime) at 4°C for 2 h. The protein band was detected using an ECL blot detection system (Azure Sapphire RGBNIR).

### 4.11 Hematoxylin–eosin staining and immunofluorescence

Livers harvested from sham, model, and CAP mice were fixed in 4% paraformaldehyde at 4°C overnight and then dehydrated with graded ethanol (100, 90, and 70%). Paraffin‐embedded liver tissues were cut into 5 mm slides, dewaxed in xylene, and then rehydrated in graded ethanol.

(1) Hematoxylin–eosin (HE) staining: The slides were stained with HE according to standard procedures.
(2) IF: After antigen retrieval using critic acid buffer, the slides were blocked in 5% BSA containing 0.1% Triton X‐100. The sections were incubated with primary antibody (TNF‐α, 60291‐1‐Ig, Proteintech; anti‐NF‐κB, 66535‐1‐Ig; Proteintech) at 4°C overnight and secondary fluorescent antibody (Alexa Fluor 488 or 647; Abcam) at 37°C for 1 h. After the sections were mounted with DAPI combined with antifluorescence quencher, images were captured using a Leica TCS SP8 SR confocal fluorescence microscope.

### 4.12 Statistical analysis

Results in this study, are presented as the mean ± standard error of the mean unless described. One‐way ANOVA was used to analyze the statistical differences between three groups unless otherwise stated. Chi‐square test was used to evaluate the statistical signification in the cell proportion. Significant *p* value are indicated with asterisks as follows: *p* < 0.05, *; *p* < 0.01, **; *p* < 0.001, ***.

## AUTHOR CONTRIBUTION

JG. W., XY. L., and P. G. designed the experiments and supervised the project. Q. Z., J. L., and J. S. planned and performed the animal experiment. LL. X., JN. H., CT. Z., and CJ. F. planned and performed the animal experiment. XL. H. was performed the in vitro experiments. P. G., JY. C., JH. C., and JH. O. performed data analysis. Q. Z., P. L., F. S., and YK. W. wrote and revised the manuscript; all these authors were critically involved in manuscript preparation. The authorship order between them was assigned according to the contribution of authors during the revision period. All authors have read and approved the final manuscript.

## CONFLICT OF INTEREST STATEMENT

The authors declare no conflicts of interest.

## ETHICS STATEMENT

All animal experiments were approved by the Institutional Animal Care and Use Committee (IACUC) of the Experimental Animal Center of Shenzhen People's Hospital (Approval number: AUP‐210320‐WJG‐001‐01).

## Supporting information

Supporting Information

## Data Availability

All sequencing data are deposited in Genome Sequence Archive (GSA; https://bigd.big.ac.cn/gsa/) with the accession number of CRA010041.
