# Supplementary material for: Single‐cell RNA sequencing reveals the effects of capsaicin in the treatment of sepsis‐induced liver injury
Source: MedComm (2020). 2023 Oct 5;4(5):e395. doi: 10.1002/mco2.395 (PMC10556204; doi:10.1002/mco2.395)
Supplement: Supplementary file 1 — Supporting Information [file MCO2-4-e395-s001.pdf]

Single-cell RNA sequencing reveals the effects of capsaicin in the treatment of sepsis-induced liver injury

Qian Zhang[1,2,5,#], Jing Liu [1,#], Jing Shen[3,#], Jinhuan Ou[1,#], Yin Kwan Wong[4], Lulin Xie[1], Jingnan Huang[1], Chunting Zhang[1], Chunjin Fu[1], Junhui Chen[1], Jiayun Chen[5], Xueling He[5], Fei Shi[6,], Piao Luo[1,2,5,*], Ping Gong[7*], Xueyan Liu [1*], Jigang Wang[1,2,5,*]

1 Department of Critical Medicine, and Shenzhen Clinical Research Centre for Geriatrics, Shenzhen People's Hospital, First Affiliated Hospital of Southern University of Science and Technology, Second Clinical Medicine College of Jinan University, Shenzhen, Shenzhen 518020, Guangdong, China

2 School of Traditional Chinese Medicine and School of Pharmaceutical Sciences, Southern Medical University, Guangzhou 510515, Guangdong, China

3 Department of Oncology, Shenzhen People's Hospital, The First Affiliated Hospital, Southern University of Science and Technology, Shenzhen 518020, Guangdong, China.

4 Department of Physiology, Yong Loo Lin School of Medicine, National University of Singapore, Singapore 117600, Singapore

5 State Key Laboratory for Quality Ensurance and Sustainable Use of Dao-di Herbs, Artemisinin Research Center, and Institute of Chinese Materia Medica, China Academy of Chinese Medical Sciences, Beijing 100700, China

6 Department of Infectious Disease, Shenzhen People's Hospital, The First Affiliated Hospital, Southern University of Science and Technology, Shenzhen 518020, Guangdong, China.

7 Department of Emergency, Shenzhen People's Hospital, The First Affiliated Hospital, Southern University of Science and Technology, Shenzhen City, Guangdong Province, China.

* Corresponding authors.

E-mail addresses: jgwang@icmm.ac.cn (Jigang Wang)

13554843721@163.com (Xueyan Liu)

gongp828@sina.cn (Ping Gong)

luopiao168@163.com (Piao Luo)

# These authors made equal contributions to this work.

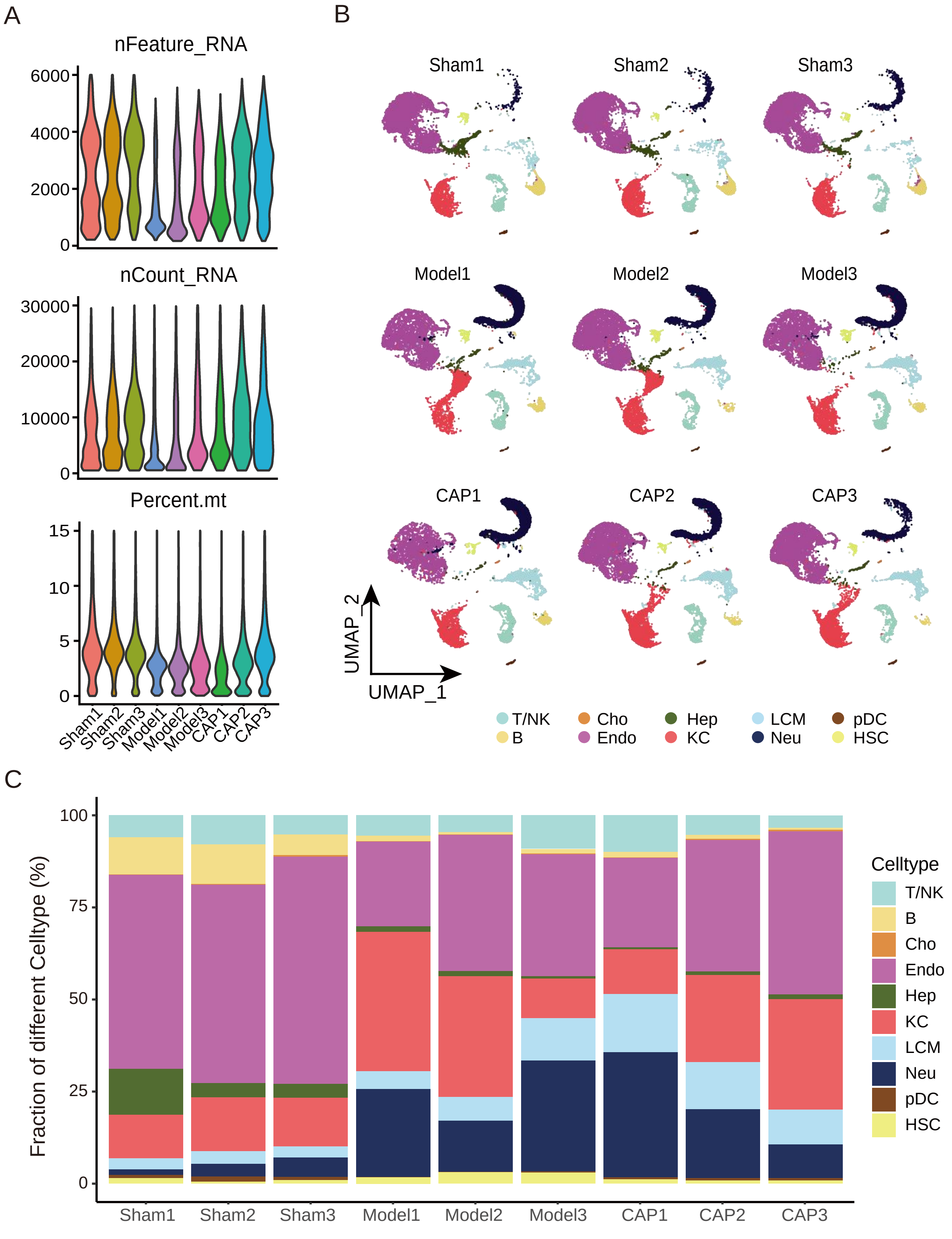

Figure S1. Landscape of intrahepatic cells from each mouse, related to Figure 1

A. Violin plots showing the content of quality control of each dataset by 10x Genomics, including the number of total UMI counts, the number of detected genes, and the proportion of mitochondrial gene counts.

B. Discrete UAMP plots showing profiles of cell compositions across each murine liver.

C. Bar chart displaying the fractions of ten liver cell clusters of each mouse.

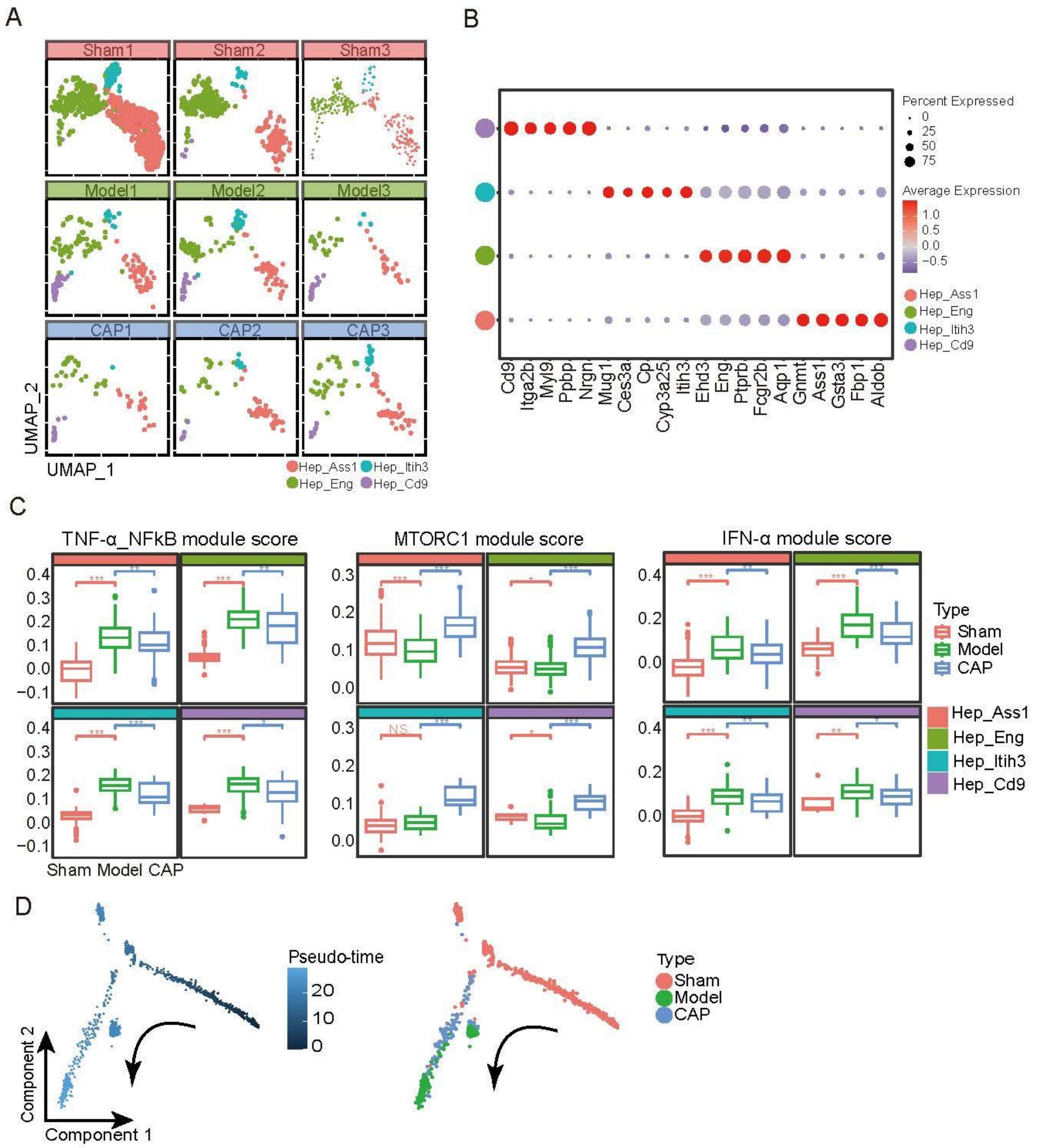

Figure S2. Gene expression analysis for hepatocyte subsets, related to Figure 2

A. UMAP plot for each dataset of mouse liver, showing the major subpopulations of hepatocytes (Hep_Ass1, Hep_Eng, Hep_Itih3 and Hep_Cd9), characterized with different colors.

B. Bubble map showing marker gene expression ranked top 5 from each hepatocyte subset.

C. Boxplot showing the TNF-α_NFkB, mTORC1 and IFN-α expression score associated with inflammation for all hepatocyte subpopulations across Sham, Model, CAP. The whiskers indicate the data scale. Groups were compared by wilcox-test. (* $p < 0.05$; ** $p < 0.01$; *** $p < 0.001$).

D. Pseudotime trajectory plot with Monocle2 depicted to reveal the development of hepatocytes cells. Each color represents the dispersion according to pseudotime and type, respectively.

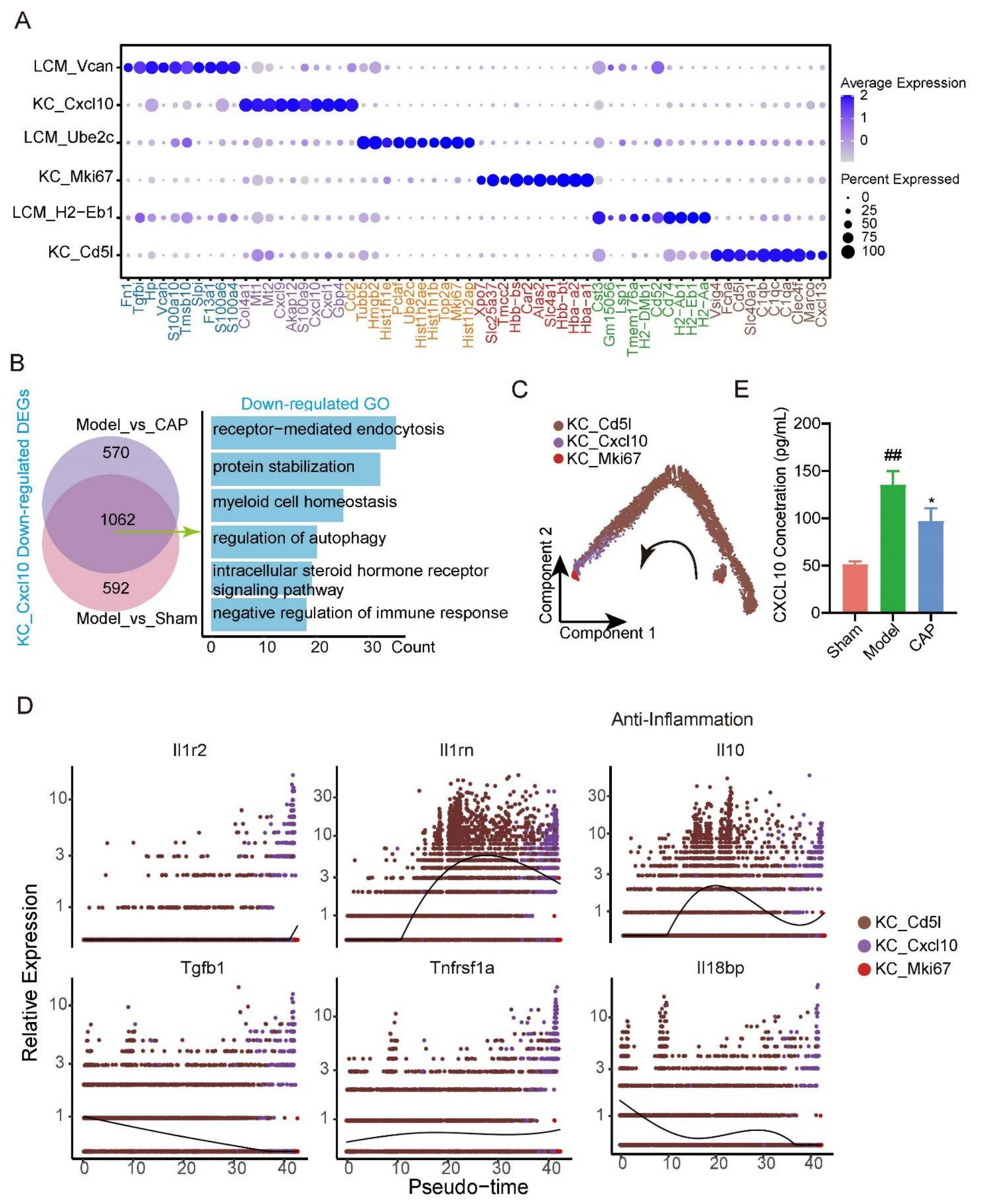

Figure S3 Gene expression and enrichment analyses for macrophage subsets, related to Figure 3

A. Bubble map showing expression of marker genes ranked top 10 from each macrophage subset.

B. Venn Diagrams showing down-regulated differential genes of KC_Cxcl10 of pairwise comparison (Model vs Sham and Model vs CAP). Histogram showing GO enrichment for commonly down-regulated genes of KC_Cxcl10 from pairwise comparison.

C. Pesudotime analysis of all Kupffer cell subtypes implemented by Monocle2, indicating the development of Kupffer subtypes.

D. Dynamic expression patterns of anti-inflammatory genes along the pseudotime, including Il10, Il18bp, Il1r2, Il1rn, Tgfb1 and Tnfrsf1a.

E. The expression level of Cxcl10 on tissue by ELISA assay (mean ± SEM, n = 3; ## $p < 0.01$ vs Sham, * $p < 0.05$ vs Model).

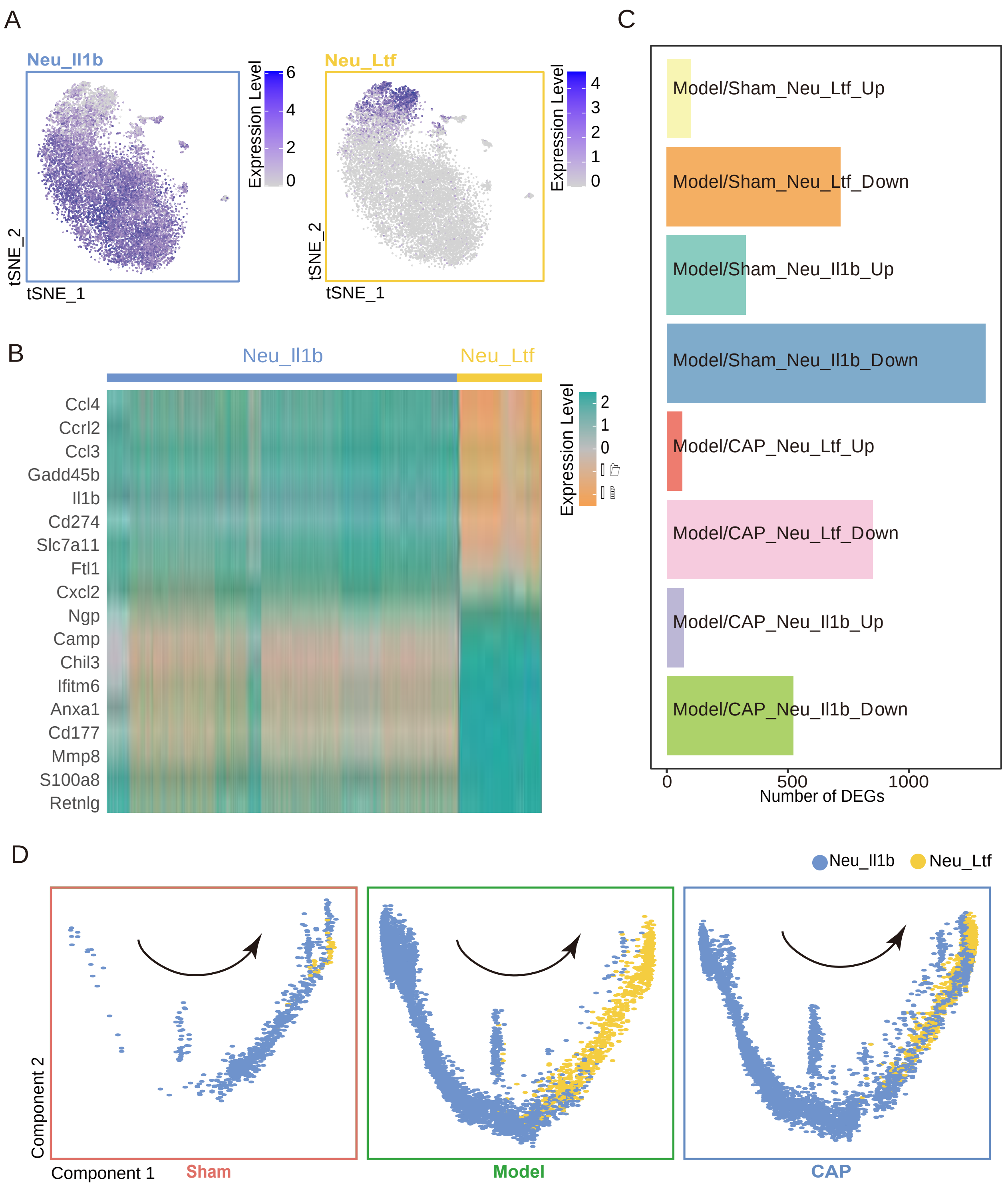

Figure S4. Gene expression and enrichment analyses for neutrophil subtypes, related to Figure 4

A. T-SNE plots showing expression patterns of characteristic genes Ltf (left) and Il1b (right) in neutrophils.

B. Heatmap showing top 20 expressed marker genes from Neu_Il1b and Neu_Ltf.

C. Bar chart showing the number of up and down-regulated genes of each neutrophil subtype from respective comparison Model vs Sham and Model vs CAP.

D. Pesudotime trajectory plots implemented by Monocle2 showing the developmental direction of neutrophil subtypes, labelled by the black arrowed lines.

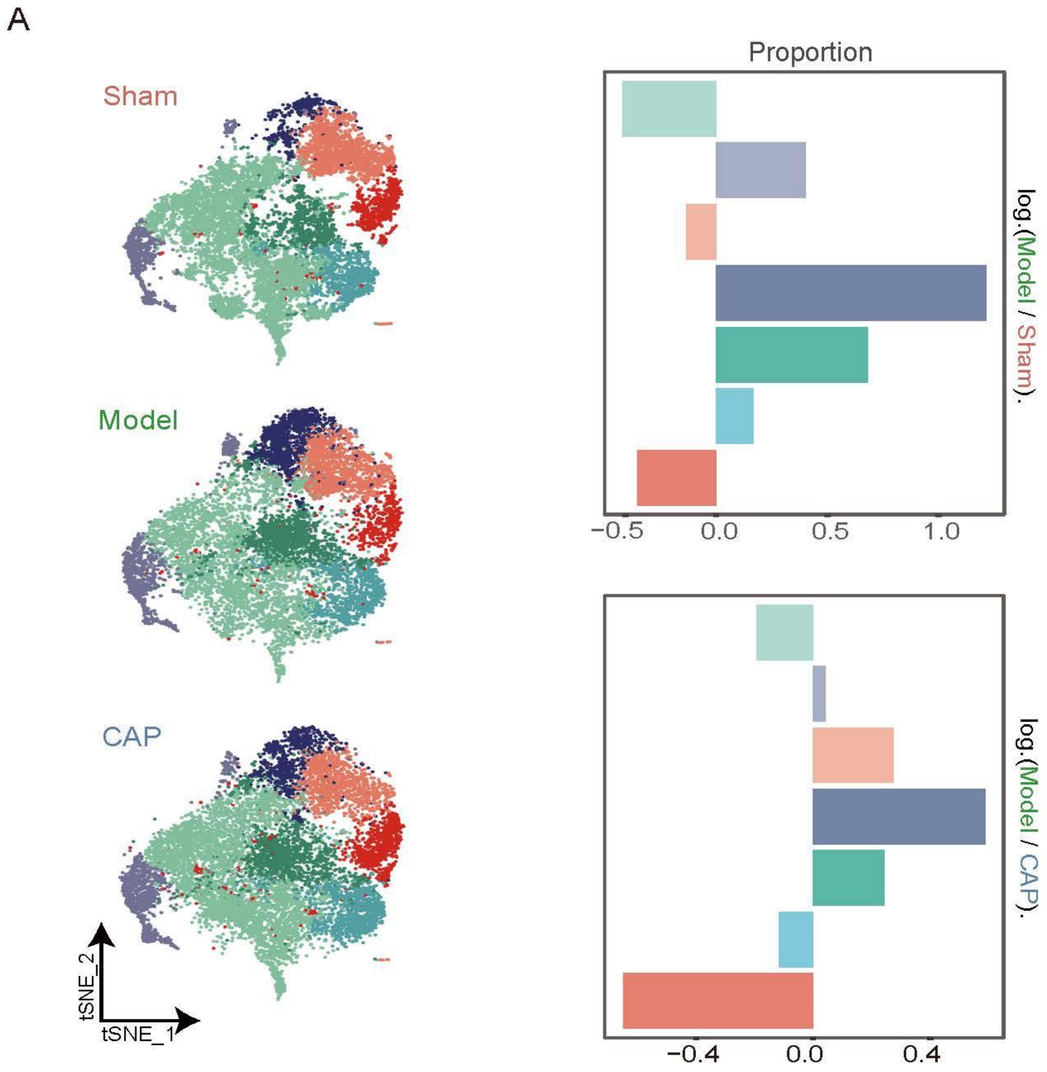

Figure S5 Characterization of endothelial cell subsets, related to Figure 5

A. T-SNE plots (left) for endothelial cells across Sham, Model and CAP. Bar chart (right) showing variation in quantity of each subtype from comparison Model vs Sham, Model vs CAP.

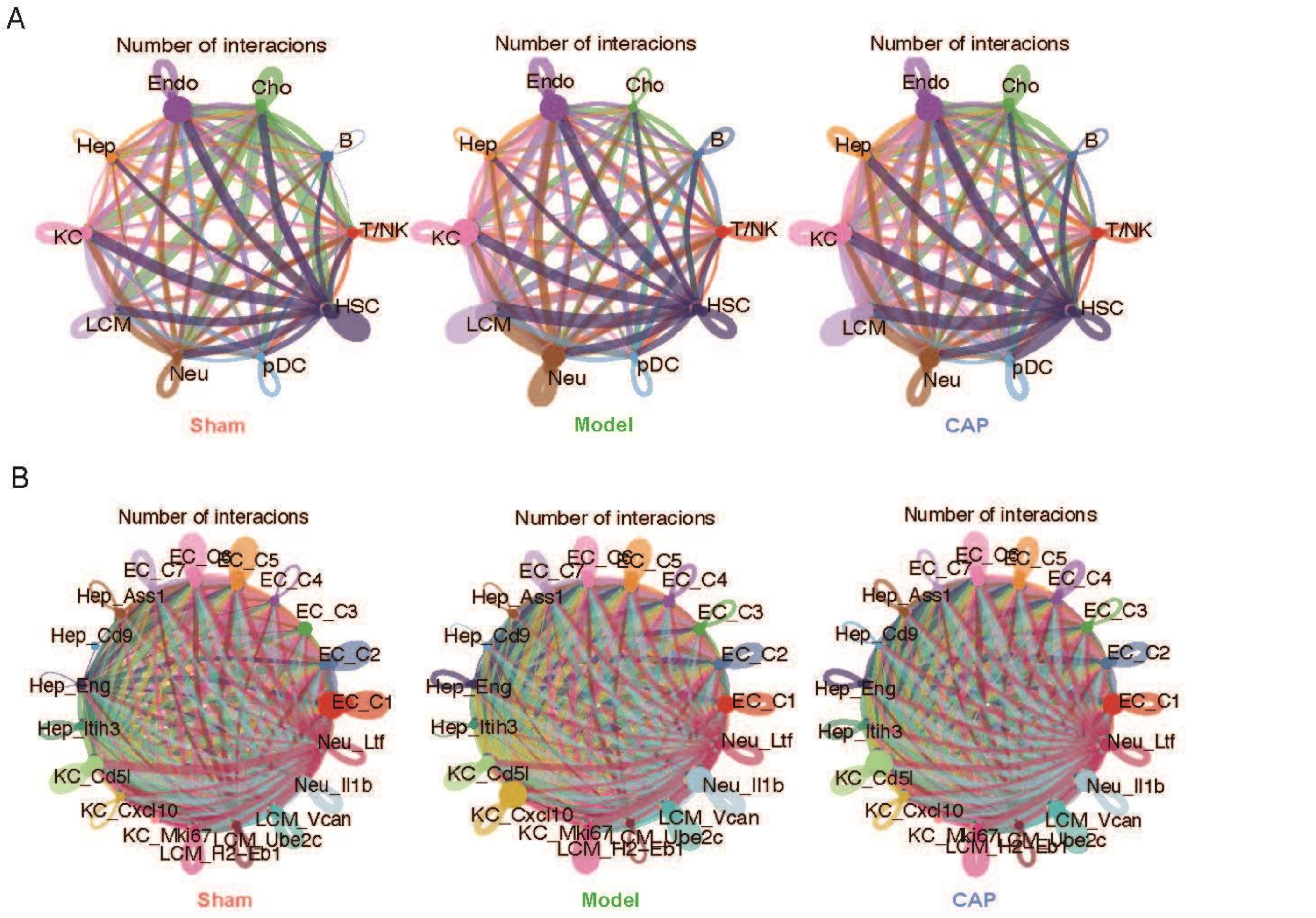

Figure S6 Predicted cell-cell interaction based on ligand-receptor pairing, related to Figure 6

A. Circle plots showing cellular interaction number across the ten major intrahepatic populations, inferred by LR pairing.

B. Circos plots showing the putative cell-cell communication of subtypes of Hep, EC, Neu and macrophages.
